# Single-operator circumcision using a penile circumcision and suturing device: a pilot comparative feasibility study

**DOI:** 10.3389/fsurg.2026.1871497

**Published:** 2026-06-25

**Authors:** Tingli Chen, Haixia Chen, Huijian Zhang

**Affiliations:** Department of Urology, Nanfang Hospital, Southern Medical University, Guangzhou, China

**Keywords:** circumcision, outpatient surgery, penile circumcision and suturing device, single-operator technique, surgical innovation, surgical workflow

## Abstract

**Background:**

Routine circumcision using penile circumcision and suturing devices (PCSD) is widely performed in outpatient settings. However, the conventional technique usually requires two operators, which increases manpower demand and limits procedural efficiency in high-volume clinical practice.

**Objectives:**

To evaluate the feasibility and short-term perioperative outcomes of a single-operator circumcision technique using a penile circumcision and suturing device, compared with the traditional dual-operator method.

**Materials and methods:**

This prospective pilot observational study consecutively enrolled patients undergoing circumcision at a single center. Patients were divided into two groups: Group 1 (dual-operator circumcision, *n* = 82) and Group 2 (single-operator circumcision, *n* = 113). All patients received the same anesthesia protocol consisting of 2% lidocaine for dorsal penile nerve block (DPNB). Operative time, intraoperative blood loss, and adverse surgical events were compared between the two groups.

**Results:**

The mean operative time was 6.18 ± 0.22 min in the single-operator group and 6.14 ± 0.25 min in the dual-operator group, with no statistically significant difference (*P* = 0.295). The mean difference was 0.04 min (95% confidence interval, −0.03–0.11). The mean intraoperative blood loss was 4.95 ± 2.41 cm² in the single-operator group and 5.01 ± 2.90 cm² in the dual-operator group, with no statistically significant difference (*P* = 0.864). The mean difference was −0.06 cm² (95% confidence interval, −0.78–0.66). No adverse surgical events occurred in either group.

**Conclusion:**

This pilot comparative study suggests that single-operator circumcision using a penile circumcision and suturing device is feasible and associated with comparable short-term perioperative outcomes. However, the present study was not designed as a formal equivalence or non-inferiority trial, and the absence of statistically significant differences should not be interpreted as proof of equivalence. Further studies are required to confirm safety, long-term outcomes, and potential resource implications.

## 1 Introduction

Circumcision is a standard intervention for phimosis and redundant prepuce (1, 2). Minimally invasive approaches using penile circumcision and suturing devices (PCSD) have gained widespread acceptance because of their advantages in reducing operative time and postoperative complications (3–5). However, traditional PCSD procedures usually require two surgeons working in coordination, which reduces procedural efficiency and increases dependence on human resources, particularly in high-volume clinical settings (3, 4).

With the increasing demand for outpatient and day-case surgery, optimizing surgical workflows and reducing unnecessary manpower consumption have become important goals (6, 7). To address these challenges, a single-operator circumcision technique based on PCSD was developed and applied. The present study prospectively compares the feasibility and short-term perioperative outcomes of this single-operator technique with the conventional dual-operator method.

## 2 Materials and methods

### 2.1 Study design and patients

This was a prospective pilot observational comparative study conducted in an outpatient setting. Patients presenting to the outpatient clinic and requesting circumcision between July 5, 2025 and November 5, 2025 were consecutively screened for eligibility. Written informed consent was obtained from all participants prior to surgery. All participants were adults (≥18 years old). The study protocol was reviewed and approved by the Medical Ethics Committee of Nanfang Hospital, Southern Medical University (Approval No. NFEC-2025-318) and conducted in accordance with the Declaration of Helsinki (8).

As part of routine preoperative assessment, all patients underwent evaluation for bleeding risk, including medical history and basic coagulation testing, in accordance with published recommendations for safe circumcision practice (9, 10). Patients with abnormal coagulation profiles were not scheduled for surgery and were therefore not included in the study; a total of four patients were excluded for this reason.

Eligible patients were allocated to either the dual-operator or single-operator group according to the surgical session in routine clinical practice. Patients who scheduled surgery in the morning underwent dual-operator procedures, in which Tingli Chen served as the primary surgeon and Huijian Zhang assisted. Patients who scheduled surgery in the afternoon underwent single-operator procedures performed entirely by Tingli Chen, as Huijian Zhang was unavailable due to other clinical duties. No scrub nurse, circulating nurse, anesthesia nurse, or other surgical personnel were present during the procedures. Therefore, in the single-operator group, Dr. Tingli Chen independently performed all procedures, whereas in the dual-operator group the operative team consisted solely of Dr. Tingli Chen and Dr. Huijian Zhang. The allocation was not randomized, as group assignment was determined by surgical session (morning vs afternoon), representing a form of temporal allocation. This may introduce potential chronological bias related to temporal differences in case scheduling.

All procedures were performed in an outpatient setting, and patients were discharged on the same day after surgery. A dedicated emergency response team, consisting of senior surgeons, nursing staff, and emergency equipment, was available on standby throughout the study period. In the event of intraoperative bleeding, anesthetic reactions, syncope, or other unexpected complications, immediate assistance could be provided. In total, 82 patients were included in the dual-operator group and 113 patients in the single-operator group. Both surgeons had more than five years of experience in circumcision procedures prior to the study, and their surgical techniques were considered stable, suggesting that the influence of a learning curve was likely limited; however, potential chronological or operator-related bias cannot be completely excluded.

**Figure 1:**
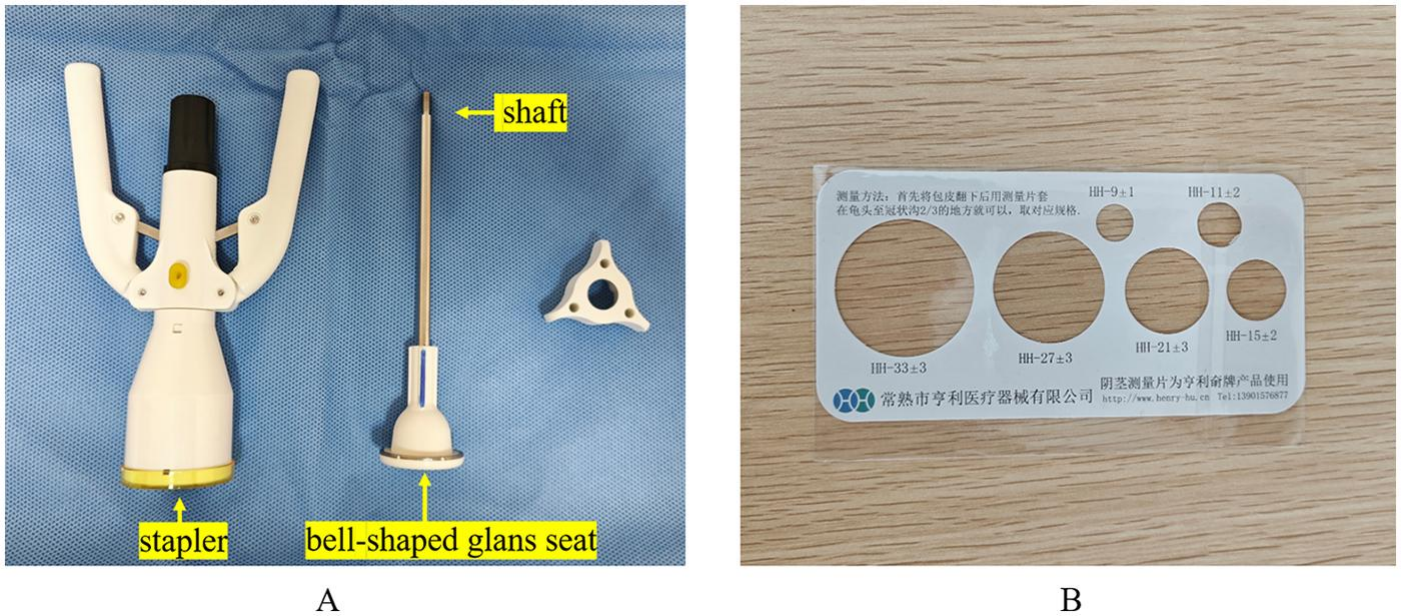
PCSD and sizing card. **(A)** PCSD (penile circumcision and suturing devices), showing the stapler component, shaft, and bell-shaped glans seat. **(B)** Sizing card used to select the appropriate device size.

The PCSD (Changzhou Henry Medical Device Co., Ltd., patent No. ZL201110130054.6; registration No. 20172020928) was used in all cases. Device models (HH-15 ± 2, HH-21 ± 3, HH-27 ± 3) were selected using a sizing card (Figures 1A,B).

**Figure 2:**
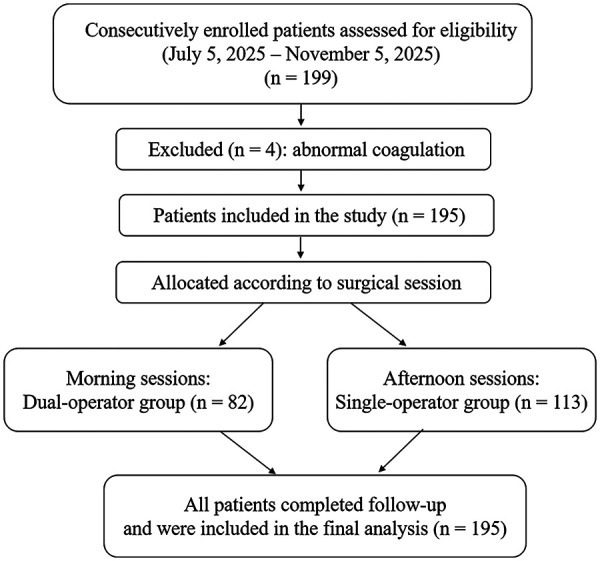
Flow diagram of patient screening, enrollment, allocation, and follow-up. Consecutively enrolled patients presenting for circumcision between July 5, 2025 and November 5, 2025 were assessed for eligibility (*n* = 199). Four patients were excluded due to abnormal coagulation profile. Eligible patients (*n* = 195) were allocated to the dual-operator or single-operator group according to surgical session (morning vs afternoon). All patients completed follow-up and were included in the final analysis.

A flow diagram illustrating patient enrollment, exclusions, allocation, and follow-up is provided in Figure 2.

### 2.2 Anesthesia protocol

All patients in both groups received the same anesthesia protocol consisting of dorsal penile nerve block (DPNB) using 2% lidocaine. DPNB was performed by injecting 2% lidocaine at the 10 and 2 o'clock positions at the base of the penis, followed by additional ventral infiltration at the 6 o'clock position. The total volume of lidocaine administered was 3–5 mL per patient. No sedation was administered during the procedure, and no other anesthetic agents or techniques were used (11, 12).

### 2.3 Surgical procedures

#### Dual-operator circumcision (traditional technique)

**Figure 3:**
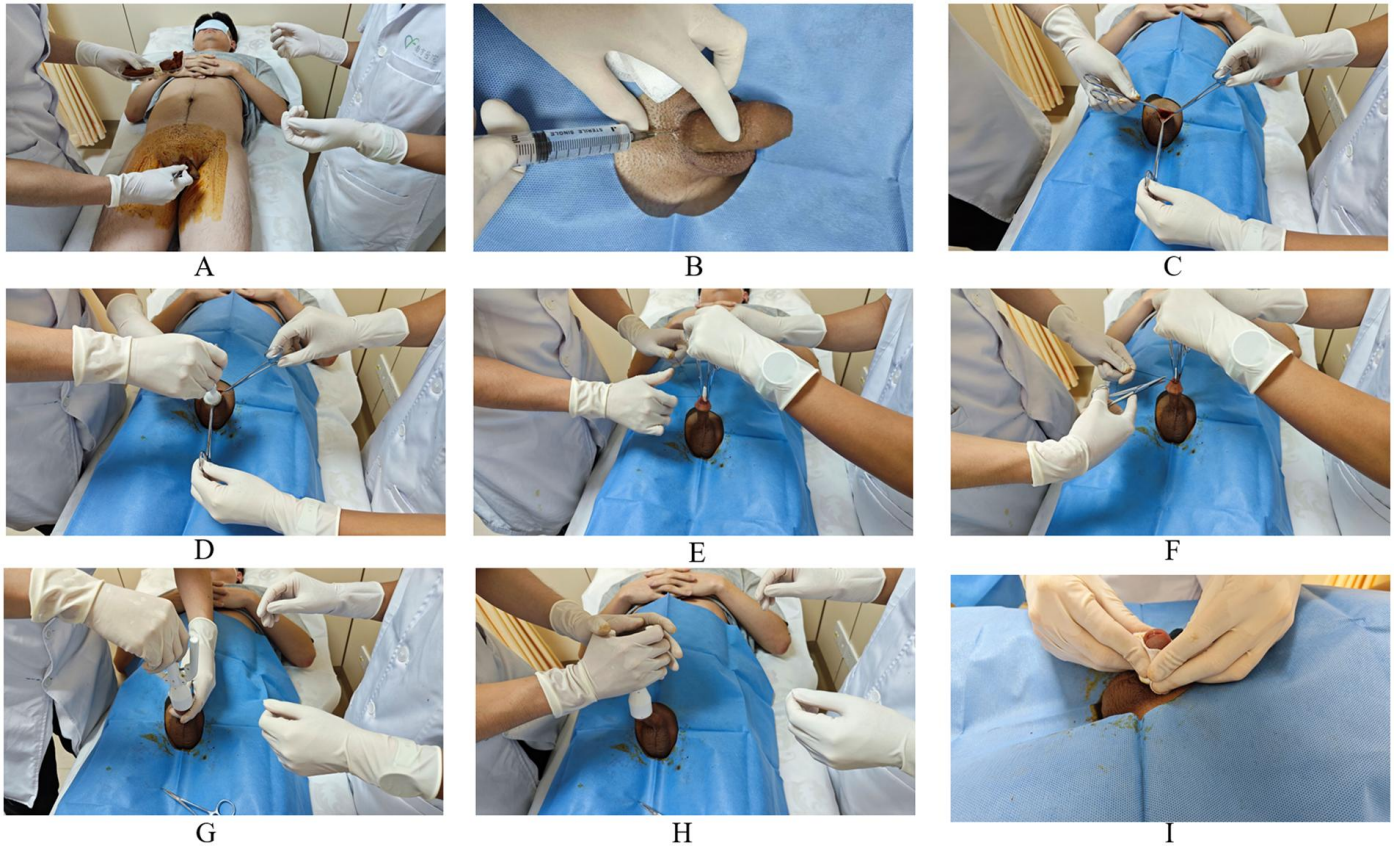
Dual-operator circumcision (traditional technique). **(A)** Disinfect the surgical field and apply sterile drapes. **(B)** Local anesthesia. **(C)** The surgeon and assistant cooperate to dilate the preputial orifice using hemostats. **(D)** The assistant holds the hemostats at the 2 and 6 o'clock positions, while the surgeon's left hand holds the 10 o'clock hemostat. With the right hand, the surgeon introduces the bell-shaped glans seat into the dilated preputial opening. **(E)** The assistant gathers the three hemostats together with the shaft of the circumcision device (as illustrated in Figure 1A), while the surgeon secures the prepuce to these instruments with silk sutures. **(F)** The surgeon cuts the sutures. **(G)** Attach the stapler component of the circumcision device (as defined in Figure 1A) to the prepositioned bell-shaped glans seat. **(H)** Activate the PCSD to excise the prepuce. **(I)** Remove the device and apply gauze compression to the incision site for hemostasis.

The traditional dual-operator circumcision was performed as previously described (2, 5). Briefly, after surgical disinfection, draping, and local anesthesia, the surgeon and assistant cooperatively dilated the preputial orifice using hemostats. The assistant held the hemostats at the 2 and 6 o'clock positions, while the surgeon manipulated the 10 o'clock hemostat. The bell-shaped glans seat was inserted, the prepuce was secured with sutures, and the PCSD was attached and activated to excise the prepuce. After device removal, gauze compression was applied for hemostasis (Figure 3).

#### Single-operator circumcision (novel technique)

After surgical disinfection and sterile draping, DPNB using 2% lidocaine was administered. A rubber tourniquet was applied at the penile root to achieve temporary hemostasis.

Three hemostats were placed at the 2, 6, and 10 o'clock positions of the preputial margin for positioning. A longitudinal incision was then made at the 12 o'clock position of the prepuce, extending approximately 2 cm distal to the coronal sulcus.

A traction suture was placed 1 cm distal to the apex of the incision. Using the left hand, the surgeon pulled the traction suture into a W-shaped configuration, thereby achieving stable exposure of the operative field without the need for an assistant. During this step, all three hemostats remained naturally suspended and were not used for traction.

The bell-shaped glans seat was inserted with the surgeon's right hand and positioned between the traction suture and the apex of the longitudinal incision. The prepuce was then gathered along the shaft and secured using a rubber band. The glans seat was carefully adjusted to ensure parallel alignment with the coronal sulcus.

**Figure 4:**
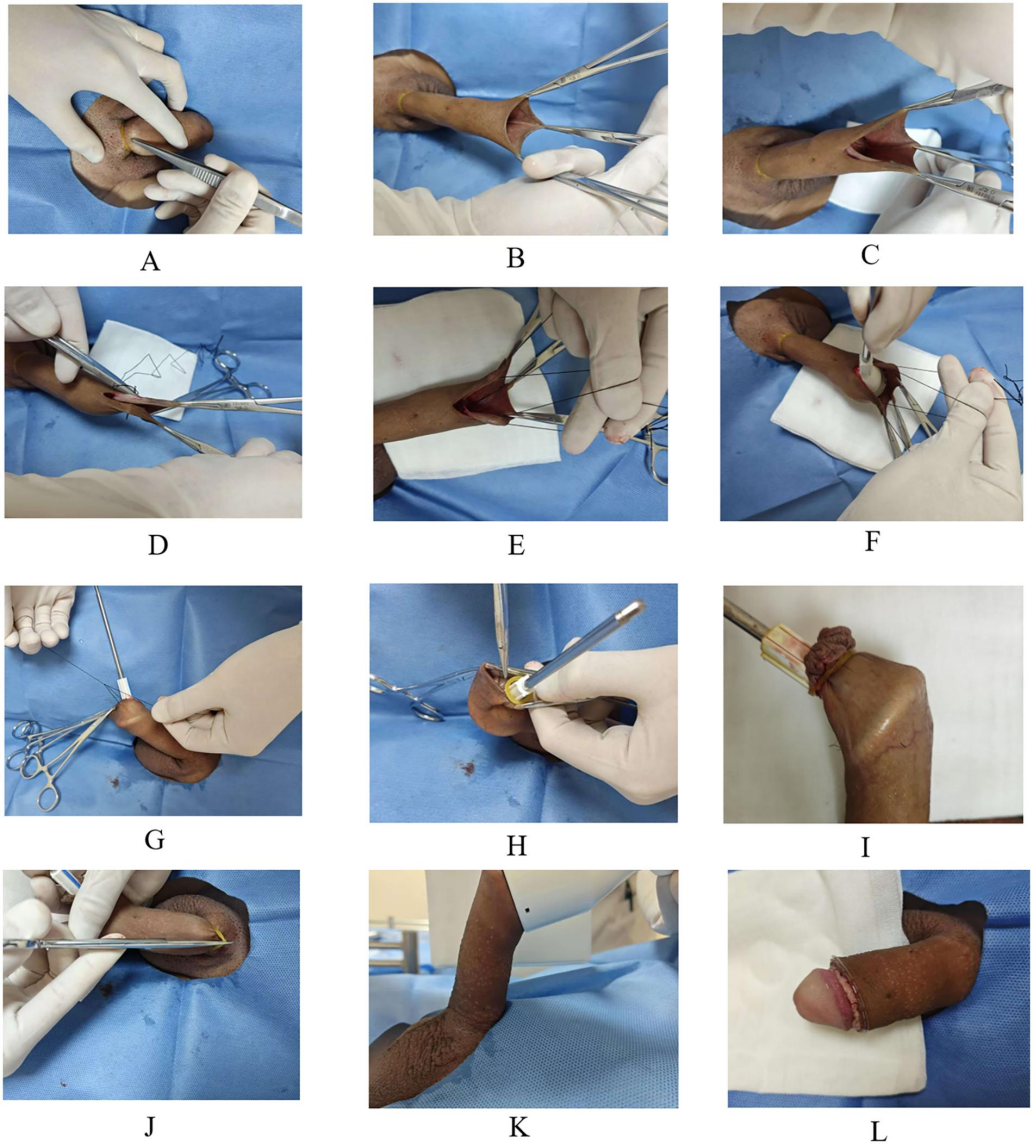
Single-operator circumcision (novel technique). **(A)** Apply a rubber tourniquet to the penile root for hemostasis. **(B)** Clamp the prepuce at the 2, 6, and 10 o'clock positions with three hemostats. **(C)** With the left hand holding the hemostats at 2 and 10 o'clock and the 6 o'clock hemostat left to hang freely, make a longitudinal incision at the 12 o'clock position using scissors in the right hand, extending the incision to 2 cm distal to the coronal sulcus. **(D)** Place a suture 1 cm distal to the apex of the longitudinal incision. **(E)** With all three hemostats left to hang freely, the surgeon applies traction by holding the suture in a “W gesture” with the left hand. **(F)** Using the right hand, the bell-shaped glans seat is then inserted precisely between the traction suture and the apex of the incision. **(G)** Tie the traction thread. **(H)** Gather the prepuce around the shaft and secure it with a rubber band. **(I)** Adjust the glans seat parallel to the coronal sulcus. **(J)** Remove the penile rubber tourniquet. **(K)** Attach the stapler component of the circumcision device to the prepositioned bell-shaped glans seat and activate the PCSD to excise the prepuce. **(L)** Remove the device and apply gauze compression to the incision site for hemostasis.

After removal of the penile tourniquet, the PCSD was attached to the prepositioned glans seat and activated to excise the prepuce. The device was subsequently removed, and gauze compression was applied to the incision site for hemostasis (Figure 4).

### 2.4 Observation indicators

The following outcome measures were recorded:

(1) Operative time: From completion of anesthesia to prepuce excision.
(2) Intraoperative blood loss: Estimated using a semi-quantitative method based on the bloodstain area on standard 8-layer sterile surgical gauze. After removal of the circumcision device, the area of blood staining on the gauze was measured in square centimeters (cm²) with the gauze laid flat under standardized lighting conditions. This method was used to provide a practical estimation of blood loss when the absolute volume was too small for accurate volumetric measurement, consistent with commonly used visual estimation approaches based on surgical gauze in clinical practice; however, it should be considered a semi-quantitative and approximate measure (13).
(3) Adverse surgical events: Including postoperative bleeding requiring reoperation, surgical site infection, or revision surgery (14, 15). Adverse surgical events were prospectively assessed by the treating clinicians during scheduled postoperative follow-up visits. All patients were routinely required to return to the outpatient clinic for postoperative wound care, and follow-up assessments were conducted concurrently as part of this standard clinical pathway. Follow-up was performed on postoperative days 1, 3, 5, and 7 for wound care and early complication assessment, and at 1 month after surgery to evaluate overall recovery. At each visit, patients were evaluated for bleeding, infection, and other wound-related complications based on clinical examination. All patients completed follow-up. No patients missed any scheduled follow-up assessment on postoperative days 1, 3, 5, 7, or 30.

### 2.5 Baseline characteristics

Baseline demographic and clinical characteristics of the two groups were collected, including age, diagnosis (phimosis or redundant prepuce), and comorbidities (diabetes mellitus and hypertension).

### 2.6 Statistical analysis

Statistical analyses were performed using SPSS 23.0 software. Continuous variables are presented as mean ± standard deviation and were compared using independent-samples t-tests. Levene's test was applied to assess homogeneity of variances. Categorical variables were compared using chi-square tests. A two-sided *P*-value ≤ 0.05 was considered statistically significant.

## 3 Results

### 3.1 Baseline characteristics

The dual-operator group included 82 patients with a mean age of 27.3 ± 6.1 years, including 39 cases of phimosis and 43 cases of redundant prepuce. There were 2 patients with diabetes mellitus and 5 with hypertension. The single-operator group included 113 patients with a mean age of 26.8 ± 5.9 years, including 60 cases of phimosis and 53 cases of redundant prepuce. There were 5 patients with diabetes mellitus and 4 with hypertension. Baseline characteristics were comparable between the two groups (Table 1).

**Table 1:** Baseline characteristics and surgical outcomes.

Variable	Dual-operator (*n* = 82)	Single-operator (*n* = 113)	*P* value
Age (years)	27.3 ± 6.1	26.8 ± 5.9	0.567
Diagnosis			0.445
Phimosis, n	39	60	
Redundant prepuce, n	43	53	
Diabetes mellitus, n	2	5	0.701
Hypertension, n	5	4	0.496
Operative time (min)	6.14 ± 0.25	6.18 ± 0.22	0.295
Blood loss (cm²)	5.01 ± 2.90	4.95 ± 2.41	0.864

### 3.2 Operative time

The mean operative time was 6.18 ± 0.22 min in the single-operator group and 6.14 ± 0.25 min in the dual-operator group, with no statistically significant difference (*P* = 0.295). The mean difference was 0.04 min (95% confidence interval, −0.03–0.11) (Table 1).

### 3.3 Intraoperative blood loss

The mean intraoperative blood loss was 4.95 ± 2.41 cm² in the single-operator group and 5.01 ± 2.90 cm² in the dual-operator group, also with no statistically significant difference (*P* = 0.864). The mean difference was −0.06 cm² (95% confidence interval, −0.78–0.66) (Table 1).

### 3.4 Adverse surgical events

No adverse surgical events, including postoperative bleeding, infection, or revision surgery, were observed in either group.

## 4 Discussion

The present study suggests that a novel single-operator circumcision technique based on PCSD can achieve perioperative outcomes (operative time, intraoperative blood loss, and adverse surgical events) comparable to those of the traditional dual-operator approach, with potential advantages in reducing dependence on surgical assistants. The present study focused primarily on perioperative outcomes to assess the immediate safety and feasibility of the technique. No significant differences were observed between the two groups in operative time, intraoperative blood loss, or adverse surgical events, suggesting that short-term perioperative performance was maintained under a simplified manpower configuration. The present study should be interpreted as an exploratory comparative study rather than a formal non-inferiority or equivalence trial. Therefore, the absence of statistically significant differences should not be interpreted as proof of equivalence between the two approaches.

In routine clinical practice, circumcision is widely regarded as a standardized, high-frequency outpatient procedure. However, in many medical institutions—particularly in primary hospitals and community healthcare settings—shortages of trained surgical assistants often limit surgical throughput (16). With the increasing demand for outpatient and day-case surgery, optimizing surgical workflows and reducing unnecessary manpower consumption have become important goals (17). The conventional PCSD technique typically requires an assistant to provide stable traction and exposure during bell-shaped glans seat placement and device fixation. This manpower dependency becomes a practical bottleneck under high case-load conditions. The single-operator technique proposed in this study addresses this limitation by reconstructing the traction mechanism through a longitudinal preputial incision combined with a traction suture and controlled hand positioning, allowing a single surgeon to complete all critical operative steps without assistance.

From a technical perspective, the key feature of the present technique lies in the establishment of a stable, assistant-independent exposure system. Through a preputial longitudinal incision and a strategically placed traction suture, sufficient and controlled exposure of the operative field can be achieved without external assistance. The temporary application of a penile tourniquet further provides effective hemostasis, compensating for potential bleeding risks associated with routine incision (18, 19). The feasibility of these operative steps is supported by the absence of increased blood loss or adverse surgical events observed in the single-operator group.

In addition to its technical feasibility, the single-operator technique presents important implications for medical resource utilization. Under conventional practice, one surgeon and one assistant are typically required for each circumcision procedure. The adoption of a single-operator model may reduce manpower requirements for this high-volume procedure. In outpatient or day-surgery units with limited staffing, such a reduction in manpower dependence may potentially improve surgical throughput and scheduling flexibility. However, these potential advantages were not directly measured in the present study and should be interpreted as organizational implications rather than confirmed outcomes. Formal evaluation of resource utilization, including staffing requirements and cost-effectiveness, warrants further investigation in future studies.

In the present study, intraoperative blood loss was estimated using a semi-quantitative gauze-area method rather than conventional volumetric measurement, as the actual blood volume during PCSD-based circumcision is usually too small for accurate collection. Visual estimation of blood loss using surgical gauze has been widely applied in surgical practice, although its accuracy is recognized to be limited (13). The comparable bloodstain areas observed between the two groups suggest that no observable difference in operative bleeding was detected between the two groups under the conditions of this study.

Several limitations of this study should be acknowledged.

First, this was a single-center study, and further validation in multicenter settings is warranted.

Second, the follow-up period was limited to early perioperative outcomes (up to 30 days); therefore, the findings of this study should be interpreted as reflecting short-term outcomes only. In addition, patient-reported and patient-centered outcomes, including postoperative pain, wound healing time, edema, cosmetic satisfaction, device-related issues, and return to normal activities, were not systematically collected. Long-term functional and cosmetic outcomes were also not assessed. These outcomes should be incorporated in future studies.

Third, the non-randomized study design, with allocation based on surgical session, may introduce potential selection bias and chronological bias. Although all procedures were performed by the same primary surgeon, which reduces inter-operator variability, operator-related bias cannot be completely excluded.

Fourth, the sample size of the present study may be insufficient to detect differences in rare adverse events, and the absence of such events in both groups should be interpreted with caution.

Nevertheless, the present study provides a practical example of how minor technical modifications may optimize surgical workflows without compromising short-term safety. The single-operator circumcision technique may be particularly valuable in outpatient clinics, primary hospitals, and settings with limited surgical workforce, where procedural efficiency and resource allocation are important considerations.

## 5 Conclusion

Single-operator circumcision using a penile circumcision and suturing device appears to be feasible and associated with comparable short-term perioperative outcomes in this pilot study. However, this study was not designed as a formal equivalence or non-inferiority trial. These findings should be interpreted as preliminary, and further studies are warranted to confirm safety, long-term outcomes, and potential resource implications.

## Data Availability

The original contributions presented in the study are included in the article/Supplementary Material, further inquiries can be directed to the corresponding author.
